# Tumor-associated macrophages: potential therapeutic strategies and future prospects in radioresistance of cancer

**DOI:** 10.3389/fimmu.2026.1844494

**Published:** 2026-07-02

**Authors:** Tianci Zhang, Ziye Zhou, Zhicheng Wang, Yanming Yang

**Affiliations:** 1 Department of Radiotherapy, The Second Hospital of Jilin University, Changchun, China; 2 National Health Commission (NHC) Key Laboratory of Radiobiology, School of Public Health, Jilin University, Changchun, China

**Keywords:** immunotherapy, radioresistance, radiotherapy, tumor microenvironment, tumor-associated macrophages

## Abstract

Radiotherapy is a cornerstone of cancer treatment. However, radioresistance remains a major obstacle limiting its efficacy. Tumor-associated macrophages (TAMs) are essential regulatory cells within the tumor microenvironment (TME) and exert complex, context-dependent effects on radioresistance. Rather than conforming to fixed M1/M2 phenotypes, TAMs are a plastic and heterogeneous population. Their functional states are shaped by tumor type, spatial localization, radiation dose, fractionation schedule, and time post-irradiation. This review summarizes how radiotherapy dynamically remodels the functional states of TAMs via inflammatory and chemotactic signaling, hypoxia- and lactate-associated metabolic adaptation, extracellular vesicle communication, damage-associated molecular pattern release and cyclic GMP-AMP synthase–stimulator of interferon genes signaling, and altered phagocytosis and antigen processing, in a regimen- and modality-dependent manner. TAMs can promote radioresistance by enhancing DNA damage repair, maintaining cancer stem cell-like properties, inducing aberrant angiogenesis and lymphangiogenesis, remodeling the extracellular matrix, altering metabolism, and shaping an immunosuppressive TME. Conversely, under specific radiation doses, time windows, and immunological contexts, certain TAMs may enhance tumor radiosensitivity. These effects involve increasing oxidative and nitrosative stress, impairing DNA repair, promoting vascular normalization, improving tissue oxygenation, and amplifying radiotherapy-induced antitumor immunity. Finally, this review discusses TAM-targeted strategies combined with radiotherapy, including inhibiting TAM recruitment and survival, functional reprogramming, immune checkpoint blockade, TAM depletion, and macrophage-based delivery platforms. To date, evidence mostly stems from preclinical or early clinical studies, lacking direct proof that TAM-targeted interventions enhance clinical radiotherapy efficacy. Future research should define functional signatures, optimize treatment timing, and establish safety profiles to facilitate clinical translation of TAM-targeted radioimmunotherapy.

## 1 Introduction

Radiotherapy remains a cornerstone of comprehensive cancer treatment, contributing to the control and cure of most tumor types, and is applicable to up to 60% of cancer patients (1). However, radioresistance remains a critical clinical challenge leading to treatment failure and poor prognosis (2). The mechanisms underlying radiotherapy resistance are multifaceted. Intrinsic direct factors include radiation-induced DNA damage repair, cell cycle arrest, and the maintenance of cancer stem cell (CSC)-like properties (2, 3), whereas extrinsic indirect factors are primarily associated with regulation by the tumor microenvironment (TME) (3, 4).

The TME is a highly orchestrated ecosystem comprising tumor cells, cancer-associated fibroblasts (CAFs), tumor-associated macrophages (TAMs), cytotoxic T lymphocytes (CTLs), regulatory T cells (Tregs), myeloid-derived suppressor cells (MDSCs), mesenchymal stem cells (MSCs), and endothelial cells (ECs) (5). Radiotherapy can significantly remodel the TME and may play a pivotal role in radioresistance by modulating immune cell infiltration and function (4). Studies have demonstrated that in esophageal squamous cell carcinoma (ESCC), radiation-induced DNA damage responses (DDR) promote the release of cytokines and chemokines, thereby altering the TME, suppressing immune function, and facilitating tumor invasion and metastasis (6).

TAMs represent a dominant immune cell population involved in TME formation. They are primarily derived from circulating monocyte precursors and, to a lesser extent, from tissue-resident macrophages (7). TAMs participate in diverse biological processes, including phagocytosis, antigen presentation, tissue repair, and tumor progression (8). In various solid tumors, TAMs and their precursors constitute a substantial proportion of tumor-infiltrating cells, accounting for up to 50% in some cases, making them a critical component of the TME that influences tumor immune status and therapeutic outcomes (9). TAMs secrete a variety of signaling molecules, including interleukin-10 (IL-10), transforming growth factor-β (TGF-β), vascular endothelial growth factor (VEGF), and matrix metalloproteinases (MMPs). These factors facilitate intricate crosstalk with multiple cellular components within the TME, such as tumor cells, CD4^+^ T cells, CD8^+^ T cells, Tregs, neutrophils, natural killer (NK) cells, and CAFs. Ultimately, this cellular network fosters tumor growth, angiogenesis, metastasis, and immunosuppression, thereby conferring resistance to conventional treatments like radiotherapy and chemotherapy (8, 9).

Traditionally, TAMs have been simply categorized into two functionally opposing subtypes: classically activated M1 macrophages and alternatively activated M2 macrophages (7). Under this classification, M1 macrophages primarily exert antitumor effects by producing reactive oxygen species (ROS), reactive nitrogen species (RNS), and pro-inflammatory cytokines such as interleukin-1 (IL-1), interleukin-6 (IL-6), and tumor necrosis factor-α (TNF-α) (10). In contrast, M2 macrophages predominantly exert pro-tumorigenic effects by promoting tumor cell proliferation, neovascularization, lymphangiogenesis, epithelial–mesenchymal transition (EMT), matrix remodeling, and the maintenance of CSC-like properties (11). However, the M1/M2 binary model merely reflects two endpoints of the macrophage functional spectrum and fails to adequately capture the functional complexity of TAMs (12). For example, studies have reported that in oral squamous cell carcinoma, M1-like macrophages may also participate in the maintenance of CSC-like properties, thereby exhibiting pro-tumorigenic functions; it is therefore inappropriate to equate M1 with an antitumor functional state or M2 with a pro-tumorigenic functional state (11, 13).

In recent years, a growing body of evidence has indicated that TAMs do not simply conform to fixed M1 or M2 states, but rather exist along a dynamic continuum with multiple intermediate states. Under different tumor types and TME stimuli, TAMs exhibit diverse functional states (7, 11). Current research primarily describes and classifies TAM subtypes based on cell surface markers, transcriptomic signatures, protein expression profiles, and functional differences. Technologies such as single-cell RNA sequencing (scRNA-seq), spatial multi-omics, and immunohistochemistry have further revealed the diversity and spatiotemporal heterogeneity of TAMs (14). A review of scRNA-seq cancer studies published in major journals identified seven representative TAM functional states: interferon-primed TAMs (IFN-TAMs), immune regulatory TAMs (Reg-TAMs), inflammatory cytokine-enriched TAMs (Inflam-TAMs), lipid-associated TAMs (LA-TAMs), pro-angiogenic TAMs (Angio-TAMs), RTM-like TAMs (RTM-TAMs), and proliferating TAMs (Prolif-TAMs), which have been observed across multiple tumor types, as summarized in (15). Beyond these, additional TAM functional states with characteristic expression profiles have been reported in specific cancer types and studies. For example, these include C1q^+^ TAMs associated with complement signaling, phagocytosis, and immune regulation, and MARCO^+^ TAMs associated with immunosuppression (16, 17) (**Table 1**).

**Table 1:** Representative TAM functional states.

Functional states	Main signatures	Main functions	Potential association with radiotherapy	References
IFN-TAMs	ISG15, CXCL10, CD274, STAT1, IRF7	Early: Immune activation; Sustained IFN signaling: Immunosuppression.	Radiotherapy can induce STING-type I IFN signaling; IFN-TAMs may represent an interferon-responsive state in TAMs after radiotherapy and may be associated with immune resistance related to immune checkpoints.	(15, 122)
Reg-TAMs	ARG1, MRC1, CX3CR1,CD274, IL10, TGFB1	Immune regulation, T cell inhibition, immunosuppressive TME formation.	Low-fraction radiotherapy can promote CCL8+ macrophage infiltration and enhance the tumor-promoting function of this population; meanwhile, it can also upregulate the expression of immunosuppressive genes, mediating post-radiotherapy immunosuppression.	(15, 21)
Inflam-TAMs	IL1B, TNF, CCL3, CXCL2, CXCL3	Promotion of tumor inflammatory response, regulation of immune cells	Radiotherapy can induce immunogenic tumor cell death and acute inflammatory signals, thereby activating inflammatory and immune responses; however, the persistent remodeling of the TME may also exacerbate the accumulation of immunosuppressive myeloid cells such as TAMs and MDSCs, mediating immune resistance.	(15, 123)
LA-TAMs	APOC1, APOE, TREM2, LPL, FABP5	Lipid metabolism reprogramming, promotion of immune suppression and tumor progression	It may be involved in the metabolic adaptation of the TME after radiotherapy and the formation of an immunosuppressive niche, mediating immunosuppression.	(15, 21, 124)
Angio-TAMs	VEGFA, SPP1, MMP9, HIF1A, ANGPT2, CXCL8, TGFB	Promotion of angiogenesis, tumor metastasis, and therapeutic resistance.	Radiotherapy can promote the expression of pro-angiogenic signals such as VEGF, SDF-1, and CSF-1, recruiting and activating Angio-TAMs; these TAMs may participate in post-irradiation vascular recovery, tumor regrowth, and radioresistance.	(15, 90)
RTM-TAMs	FOLR2, LYVE1, HES1, TIMD4, IGF1, C1QA, C1QB	Maintenance of tissue homeostasis, promotion of EMT, ECM remodeling, antigen presentation, promotion of Tregs recruitment, and tumor invasion, etc.	It may be involved in processes such as tissue damage repair, fibrosis, perivascular immune regulation, and immune resistance after radiotherapy (direct evidence is limited, and specific associations need further study).	(15, 125)
Prolif-TAMs	MKI67, CDK1, TOP2A, STMN1, TYMS, CCNA2	Participation in the local expansion of TAMs and potential promotion of inflammatory responses, fibrosis, and tumor progression, with underlying mechanisms requiring further investigation.	It may be involved in the replenishment of local TAMs and radioresistance after radiotherapy (direct evidence is limited, and specific associations need further study).	(15, 126)

This table summarizes the functional states, main signatures, main functions, and potential associations with radiotherapy for seven representative TAM populations. It is important to emphasize that these functional states are not fixed or independent cell types, but rather reflect the functional tendencies of TAMs under specific TME contexts. There may be some overlap between different functional states, and these changes can vary depending on tumor type, tissue source, spatial niche, and other factors.

In summary, a unified classification framework for TAMs is currently lacking. The categorization of TAM subpopulations is substantially influenced by tumor type, tissue of origin, spatial niche, sequencing platform, and analytical strategy. This not only reflects the high degree of heterogeneity and context-dependence of TAMs but also poses significant challenges for the standardization of subpopulation classification and functional annotation, as well as for cross-study comparisons (9, 14, 15). Because many experimental studies describe macrophage states using conventional M1/M2-associated markers, this review retains the terms “M1-like” and “M2-like” when discussing these original findings. However, these terms are used only to summarize the functional tendencies of TAMs under specific experimental conditions and should not be interpreted as stable, mutually exclusive, or universally applicable TAM classifications.

As noted above, TAMs are highly plastic and context-dependent, and may exhibit either pro-radioresistance or radiosensitizing effects under different radiotherapy conditions (12). Elucidating the functional remodeling of TAMs induced by radiotherapy and the bidirectional regulatory mechanisms involved may provide novel strategies for enhancing tumor radiosensitivity and improving cancer treatment outcomes. Building on this foundation, this review focuses on the dynamic changes in TAM functional states following radiotherapy and their roles in radioresistance and radiosensitization, emphasizing that such changes are not simply M1/M2 phenotypic transitions but rather the result of complex interactions among radiation dose, fractionation schedule, time post-irradiation, tumor type, and spatial niche. Finally, this review further summarizes TAM-targeted strategies in combination with radiotherapy, translational challenges, and future directions.

## 2 Post-irradiation TAM remodeling: recruitment, functional alterations, and context-dependent responses

Post-irradiation changes in TAMs cannot be fully explained by M1/M2 polarization alone. They also involve cellular recruitment, local survival, and alterations in functional states (14, 18). These responses are influenced by tumor type, radiation dose, fractionation schedule, and post-irradiation timing. These factors may explain the inconsistent TAM changes observed across different studies (18). This section discusses dynamic changes in TAM states after radiotherapy from the perspectives of inflammatory and chemotactic signaling, metabolism and extracellular vesicle (EV) communication, damage signaling and antigen processing, and radiation dose and technical parameters.

**Figure 1:**
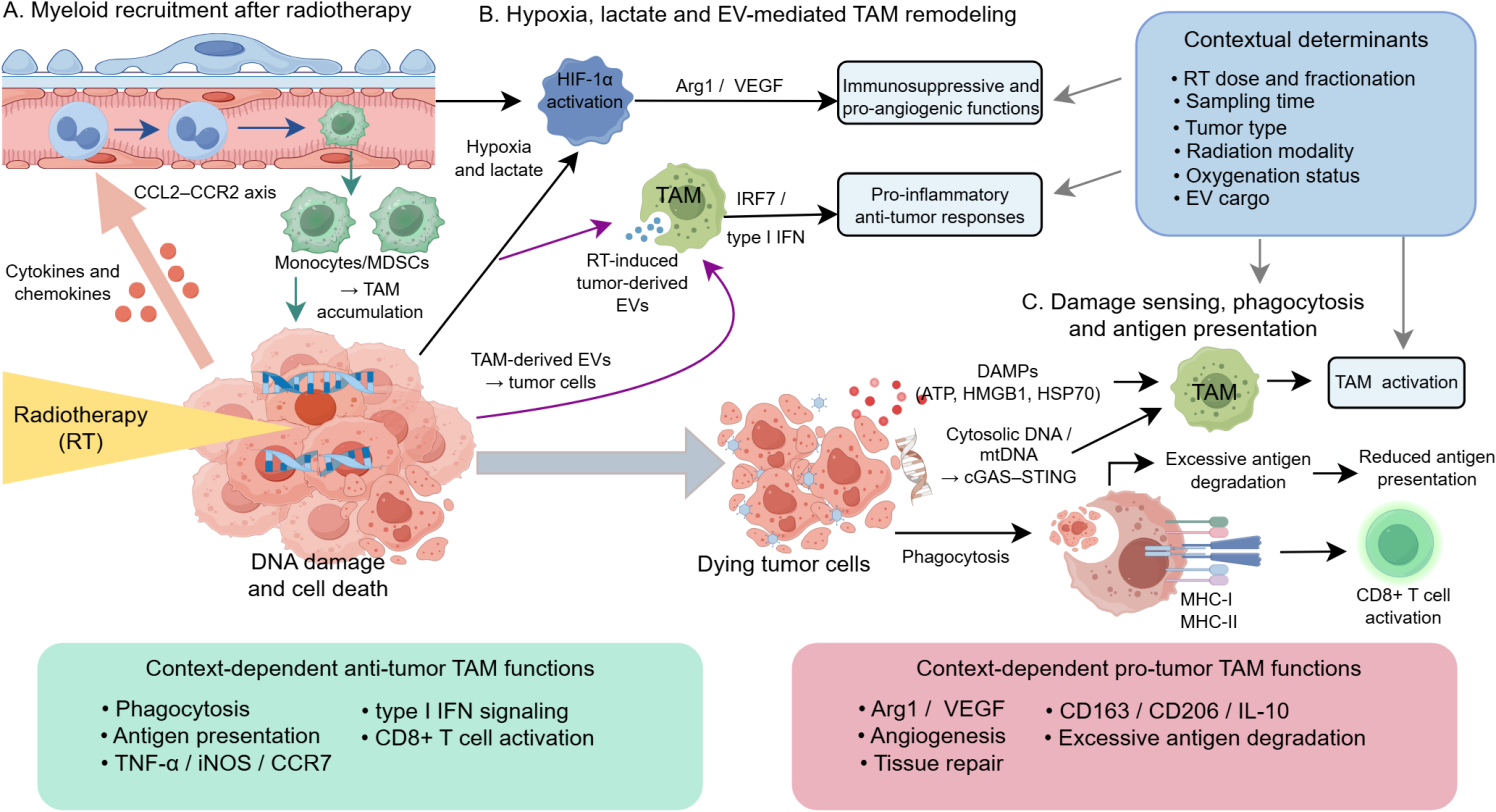
Context-dependent remodeling of tumor-associated macrophages after radiotherapy. Radiotherapy induces tumor cell DNA damage and cell death, thereby reshaping TAMs through interconnected mechanisms. **(A)** Myeloid recruitment after radiotherapy. RT-induced cytokines and chemokines, including the CCL2–CCR2 axis, promote the recruitment of MDSCs, contributing to TAM accumulation. **(B)** Hypoxia-, lactate-, and extracellular vesicle-mediated TAM remodeling. Changes in oxygenation, lactate accumulation, and EV-mediated communication can remodel TAM functions. These signals may promote HIF-1α/Arg1/VEGF-associated immunosuppressive and pro-angiogenic functions, or support IRF7/type I IFN-associated pro-inflammatory antitumor responses in specific contexts. **(C)** Damage sensing, phagocytosis, and antigen presentation. Dying tumor cells release DAMPs, tumor antigens, cytosolic DNA, and mtDNA, which can regulate TAM activation, phagocytosis, and antigen processing through innate immune sensing pathways such as cGAS–STING. Depending on antigen processing efficiency, TAMs may support MHC-associated antigen presentation and CD8^+^ T cell activation, or limit effective immunity through excessive antigen degradation. These responses are shaped by RT dose and fractionation, sampling time, tumor type, radiation modality, oxygenation status, and EV cargo.

As illustrated in **Figure 1**, post-radiotherapy TAM remodeling mainly involves three interrelated processes. These include myeloid cell recruitment, functional alterations mediated by hypoxia, lactate, and EVs, and damage sensing, phagocytosis, and antigen processing. The final functional trajectory of these reprogrammed macrophages is shaped by multiple intersecting factors, including the radiotherapy regimen, tumor context, oxygenation status, time after irradiation, and specific EV cargo.

### 2.1 Radiation-induced inflammatory signaling and TAM recruitment

Radiotherapy induces tumor cell damage and death. This process promotes the release of inflammatory mediators, cytokines, and chemokines from tumor cells and other cells within the TME (18–20). These signals recruit myeloid cells, including monocytes, macrophages, and MDSCs, into the irradiated area. They also influence the infiltration of lymphocytes, such as Tregs and tumor-infiltrating lymphocytes (TILs), thereby reshaping the local immune composition. Consequently, post-irradiation changes in TAM number or phenotype may reflect both peripheral myeloid cell recruitment and subsequent functional alterations (18, 21).

Among these chemotactic signals, the CCL2/CCR2 axis is one of the best-characterized pathways. CCL2 is produced by multiple cell types, including tumor cells, TAMs, MDSCs, MSCs, and CAFs. One of its major functions is to recruit monocytes. Radiotherapy also upregulates CCL2 expression and promotes monocyte or macrophage infiltration (19). However, the immunological consequences of CCL2 are context-dependent. For example, after 20 Gy irradiation in a sarcoma model, CCL2 upregulation was associated with Th1/Tc1 cell activation and increased infiltration. In contrast, in a head and neck squamous cell carcinoma (HNSCC) model treated with 7.5 Gy irradiation, CCL2 upregulation promoted the infiltration of TNF-α-producing monocytes and CCR2+ Tregs. This effect attenuated radiotherapy efficacy (19). These findings indicate that the role of the CCL2/CCR2 axis should be interpreted according to tumor type, radiation dose, and the specific cell types recruited.

Therefore, chemokine-mediated TAM recruitment should be regarded as an initiating step in post-irradiation TME remodeling. It should not be simply equated with an increase in pro-tumorigenic TAMs. After entering the TME, these TAMs continue to encounter additional signals, such as hypoxia, lactate, DAMPs, and EVs. Their functional states may continue to evolve thereafter (18, 20, 22).

### 2.2 Hypoxia, lactate, and extracellular vesicle-mediated functional remodeling of TAMs

Hypoxia is a common feature of the solid tumor TME. It influences radiotherapy responses and shapes TAM function (23–25). After radiotherapy, changes in local tumor vasculature, cell death, and tissue repair processes alter local oxygenation status (18, 24). Under these conditions, TAMs are influenced not only by hypoxia but also by chemokines and EVs released from tumor cells (23). Therefore, hypoxia-associated TAM changes should be understood as functional adaptations to altered local microenvironmental conditions.

Lactate is an important mediator linking tumor glycolytic metabolism to TAM functional alterations. Colegio et al. showed that tumor cell-derived lactate stabilized HIF-1α in macrophages under normoxic conditions and induced the expression of VEGF, Arginase-1 (Arg1), and certain M2-associated genes (26). The same study also demonstrated that reducing PKM2 expression in tumor cells decreased intratumoral lactate levels and Arg1 expression in TAMs. In contrast, macrophage-specific Arg1 deletion suppressed tumor growth (26). These findings suggest that lactate is more than a glycolytic byproduct. It also serves as a signaling molecule that mediates communication between tumor cells and TAMs, promoting the acquisition of pro-angiogenic and pro-tumorigenic functional states.

EVs, particularly exosomes, represent an important mode of communication between tumor cells and TAMs. EVs carry miRNAs, lncRNAs, circRNAs, proteins, and lipids, thereby transmitting signals within the TME (27, 28). In hypoxic niches, EVs act as important mediators of intercellular communication and participate in TAM functional remodeling and tumor progression. For example, in a pancreatic neuroendocrine neoplasm model, hypoxic tumor cell-derived exosomes enriched in miR-4488 were internalized by macrophages. Following uptake, these exosomes promoted the upregulation of CD163 and CD206. This phenotypic shift conferred immunosuppressive and pro-metastatic properties and ultimately facilitated hepatic metastasis (29). In a glioblastoma (GBM) study, hypoxia induced macrophages to acquire immunosuppression-associated features. EVs derived from these macrophages further promoted the EMT, migration, and invasion of GBM cells (30). These findings indicate that EVs participate not only in the regulation of TAMs by tumor cells but also in the reciprocal regulation of tumor cells by TAMs.

Notably, in the context of radiotherapy, EVs do not necessarily promote immunosuppression. In a melanoma model, macrophages engulfed post-irradiation tumor-derived exosomes. This uptake enhanced macrophage pro-inflammatory responses and CD8+ T-cell antitumor activity through IRF7/type I IFN-associated signaling (31). In cervical cancer patients, post-irradiation plasma EVs increased the expression of CCR7, TNF-α, and iNOS in macrophages, reduced the expression of CD163 and IL-10, and enhanced phagocytic capacity (32). In rectal cancer, short-course radiotherapy was also associated with enhanced pro-inflammatory markers, antigen presentation-related molecules, and phagocytic function in TAMs. *In vitro* models suggested that EVs derived from irradiated tumor cells contributed to this process (12). Therefore, the effects of post-irradiation EVs on TAMs should not be simply classified as either pro-tumor or antitumor. Instead, they should be evaluated according to tumor type, irradiation modality, and specific EV cargo.

Hypoxia, lactate, and EVs collectively influence the functional trajectory of TAMs after radiotherapy. Their effects depend on tumor type, irradiation modality, and the stage of the TME. In certain contexts, these signals enhance pro-angiogenic, immunosuppressive, and tissue repair-associated functions. Conversely, in other settings, they promote inflammatory responses, phagocytic function, and T-cell activation. Therefore, hypoxia, lactate, and EVs are best understood as microenvironmental factors that explain functional heterogeneity in TAMs after radiotherapy (18, 23, 27).

### 2.3 Regulation of TAM phagocytosis and antigen presentation by radiation-induced damage signals

Following radiation-induced DNA damage and tumor cell death, damaged tumor cells release DAMPs, tumor antigens, and DNA-associated signals. These factors provide initiating stimuli for innate immune responses within the TME (33–36). For example, DAMPs such as HMGB1, HSP70, and ATP serve as “danger signals” after tumor injury. They facilitate immune cell recognition of damaged cells and contribute to the priming of antitumor immunity (33–35). In parallel, radiation induces the cytosolic release of nuclear and mitochondrial DNA. This process activates the cGAS-STING pathway and induces type I IFN production and inflammatory signaling. These pathways subsequently participate in post-irradiation TME remodeling (36, 37).

These damage signals further modulate TAM function. Studies indicate that mitochondrial nucleic acids derived from irradiated tumor cells promote IFN-β production and enhance macrophage proinflammatory responses through STAT1-associated pathways. This finding suggests that post-irradiation DNA-associated signals regulate macrophage function (38). In addition, in the context of radiation-induced tissue injury, cGAS-STING activation influences macrophage recruitment and inflammatory states through signals such as CCL2. However, this process does not necessarily represent an antitumor effect. Instead, it contributes to inflammatory injury in normal tissues (36, 39). Therefore, DAMP release and cGAS-STING signaling primarily modulate the functional states of TAMs following radiotherapy. They should not be simply interpreted as signals that induce a single, fixed macrophage phenotype.

Importantly, these changes do not necessarily translate into effective antitumor immunity. Following radiotherapy, TAMs clear apoptotic tumor cells and acquire additional tumor antigens. Whether these antigens subsequently induce T-cell responses depends on the capacity of TAMs to process and present them. In a pancreatic cancer model, radiotherapy combined with PI3Kγ inhibition enhanced the antigen-presenting capacity of TAMs after the clearance of apoptotic cells. This combination also promoted CD8+ T-cell responses and local tumor control (40). Conversely, excessive antigen degradation following uptake by TAMs may limit antigen presentation. Inhibition of relevant proteases increases MHC-I expression and enhances CD8+ T-cell activation (41). Therefore, radiation-induced damage signals and the clearance of dying cells shape the subsequent functions of TAMs. These macrophages may support antigen presentation and T-cell activation. Conversely, they may limit effective immune responses through excessive antigen degradation (40, 41).

### 2.4 Context-dependent regulation of TAM responses by radiotherapy regimens and technical parameters

TAM responses following radiotherapy are influenced by radiation dose, fractionation schedule, and post-irradiation sampling time. Accordingly, findings across studies remain inconsistent (18, 21). These dynamic changes should not be reduced to a fixed paradigm in which low doses induce an M1-like state and high doses induce an M2-like state (18). One review categorized irradiation doses from related studies into low-dose (<1 Gy), intermediate-dose (1–10 Gy), and high-dose (>10 Gy) ranges. Based on this classification, several general trends emerge. In specific models, low-dose irradiation is associated with iNOS-related proinflammatory functions, vascular normalization, and enhanced T-cell responses. Intermediate-dose irradiation enhances macrophage phagocytosis and proinflammatory activity. Conversely, high-dose irradiation is associated with M2-like TAM infiltration, angiogenesis, and accelerated tumor growth in certain contexts (18). However, this dose-based framework primarily serves to organize existing literature. It does not imply that identical TAM responses occur across all tumor models.

Changes in TAM composition following radiotherapy do not exclusively result from the phenotypic conversion of pre-existing TAMs. Instead, they may also arise from monocyte recruitment or differential radiosensitivity among TAM subpopulations (21, 42). For example, low-dose radiotherapy enhanced monocyte chemotaxis in a glioblastoma model. This recruitment was accompanied by an increase in proinflammatory TAM features (43). Conversely, hypofractionated radiotherapy (8 Gy × 3) increased the infiltration of CCL8high M2-like macrophages in a Lewis lung carcinoma model. This regimen simultaneously reduced their antigen presentation-related features (21). In another glioblastoma study, X-ray irradiation did not directly alter experimentally defined macrophage phenotypes. Instead, it produced differential survival among distinct subpopulations. These findings suggest that post-irradiation changes in TAM composition involve a subpopulation selection process, rather than merely the reprogramming of pre-existing cells (42).

Radiation dose and technical parameters also shape damage signaling and its downstream immune consequences. At appropriate doses, radiotherapy induces type I IFN responses through cytosolic DNA accumulation and cGAS-STING pathway activation. However, single doses exceeding approximately 12–18 Gy induce TREX1, which degrades cytosolic DNA. This degradation subsequently attenuates radiation-associated immune activation (44). Beyond dose and fractionation, radiation type and dose rate also alter immune responses within the irradiated TME. Modalities such as proton therapy, carbon-ion therapy, and FLASH-RT possess distinct dose distribution, linear energy transfer, and dose-rate characteristics. Consequently, these differences elicit distinct damage responses and immune modulation (45–48). Nevertheless, these findings derive primarily from specific preclinical models. Therefore, the resulting immune consequences should be interpreted alongside tumor type, dose, fractionation schedule, sampling time, and irradiation technique (49, 50).

Ultimately, post-irradiation changes in TAMs require contextual interpretation based on the specific tumor model, irradiation regimen, and sampling time. Furthermore, it remains crucial to distinguish whether these changes originate from peripheral monocyte recruitment, radiation-induced subpopulation selection, or the functional reprogramming of pre-existing TAMs.

## 3 Multi-tiered mechanisms by which TAMs mediate radioresistance

**Figure 2:**
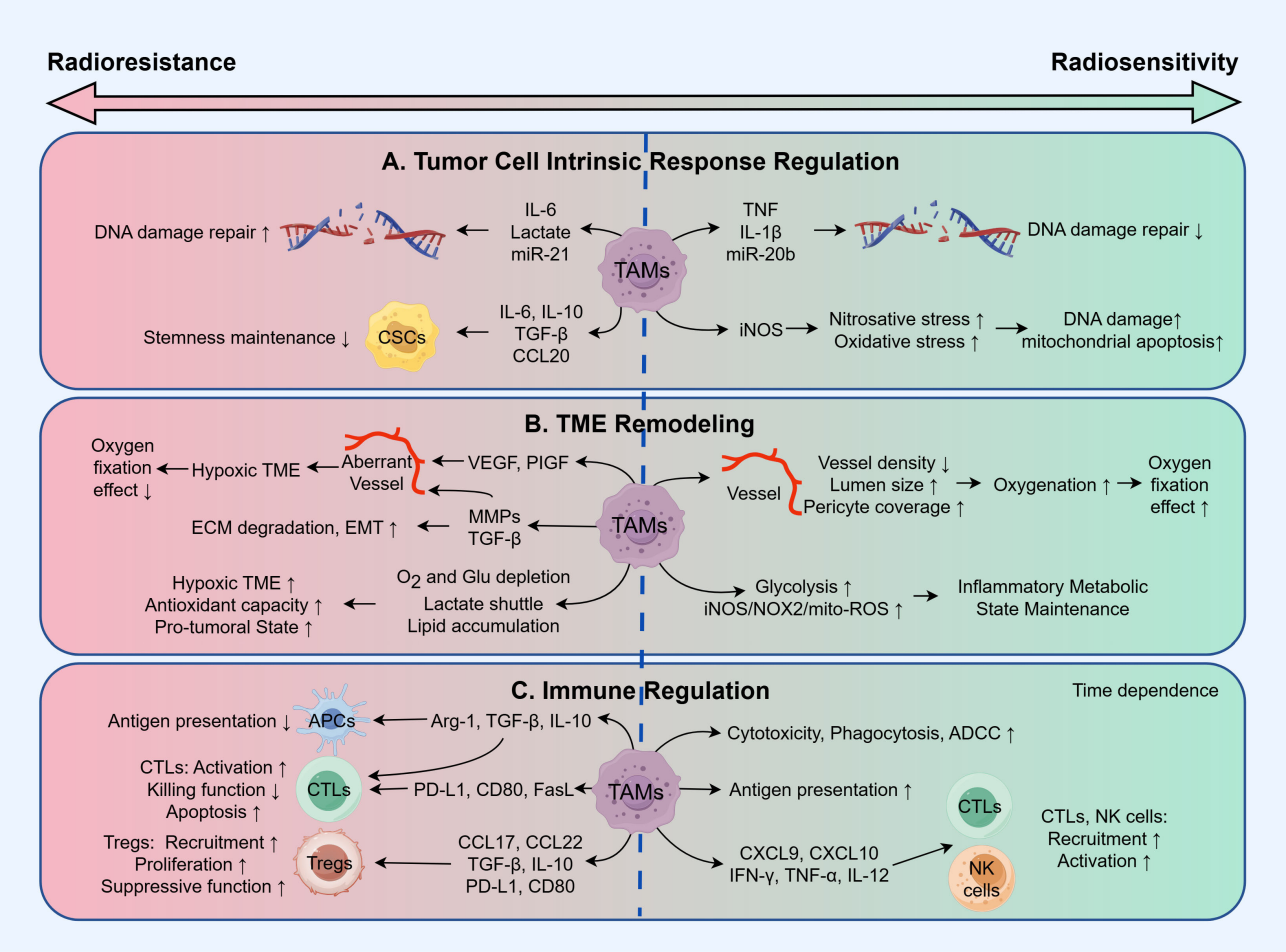
Multilevel mechanisms by which TAMs regulate tumor radioresistance and radiosensitivity. This figure summarizes the dual, context- and time-dependent roles of TAMs in shaping tumor responses to radiotherapy. The left red-shaded side depicts TAM-associated mechanisms that promote radioresistance, whereas the right green-shaded side depicts TAM-associated mechanisms that enhance radiosensitivity. These effects are organized into three interconnected layers: tumor cell-intrinsic response regulation, TME remodeling, and immune regulation. **(A)** Tumor cell-intrinsic response regulation. TAMs promote radioresistance by enhancing DNA damage repair and maintaining CSC properties. Conversely, TAMs may enhance radiosensitivity by inducing nitrosative stress, oxidative stress, DNA damage accumulation, and mitochondrial apoptosis. **(B)** TME remodeling. TAMs strengthen radioresistance by promoting aberrant angiogenesis, hypoxia, extracellular matrix remodeling, and metabolic reprogramming, thereby impairing the oxygen fixation effect. Conversely, TAMs may enhance radiosensitivity by supporting vascular normalization and improving tumor oxygenation. **(C)** Immune regulation. TAMs promote an immunosuppressive microenvironment by limiting antigen presentation, impairing CTL/NK cell function, and enhancing Treg activity, thereby contributing to radioresistance. Conversely, TAMs may enhance radiosensitivity by promoting antigen presentation and the recruitment and activation of effector immune cells.Tables.

TAM-mediated radioresistance is not the result of a single TAM subpopulation acting in isolation, but rather the collective outcome of interactions among multiple distinct TAM functional states, tumor cells, stromal cells, and immune cells (8, 9). TAMs can directly act on tumor cells to enhance DNA damage repair and maintain stemness, thereby increasing their intrinsic radioresistance. They can also indirectly mediate radioresistance by promoting aberrant angiogenesis, extracellular matrix remodeling, metabolic reprogramming, and the formation of an immunosuppressive TME (7, 14). Overall, the impact of TAMs on radiotherapy responses is highly context- and time-dependent. Their role in promoting radioresistance can be understood from three interrelated levels: tumor cell-intrinsic response regulation, TME remodeling, and immune regulation, mainly corresponding to the mechanisms shown in the red section on the left side of **Figure 2**.

### 3.1 Enhancement of intrinsic tumor cell radioresistance

#### 3.1.1 Promotion of DNA damage repair

DNA damage is the primary mechanism by which ionizing radiation (IR) kills tumor cells, including DNA strand breaks and DNA crosslinks, with double-strand breaks (DSBs) being the most critical. Extensive research demonstrates that ATM, ATR, and DNA-PKcs are key kinases involved in the repair of DSBs induced by IR and other agents. Their coordinated activity ensures that DNA damage is rapidly and accurately repaired (51). Therefore, compared to mechanisms that indirectly mediate radioresistance primarily through TME remodeling, such as aberrant angiogenesis, matrix remodeling, and immunosuppressive microenvironment formation, TAM-mediated regulation of DNA damage repair more directly influences tumor-cell responses to radiotherapy-induced injury. Thus, it is more directly linked to enhanced radioresistance.

TAMs can promote DNA damage repair through paracrine secretion of various signaling molecules and metabolic intermediates. For example, in prostate cancer, TAMs can secrete IL-6. This cytokine transcriptionally upregulates DNA damage repair factors such as ATM and ATR and activates ATM. In glioblastoma, TAMs can release lactate through monocarboxylate transporter 4 (MCT4). This lactate can induce lactylation of Ku70 at lysine 317, promoting the formation of the Ku70–Ku80–DNA-PKcs complex, enhancing non-homologous end joining (NHEJ), and thereby facilitating DSB repair (52, 53). Exosome-mediated miRNA transfer may represent another mode of paracrine regulation of the DDR by TAMs. Studies have shown that miR-21 can target PTEN or PDCD4. This activates PI3K/Akt/mTOR and cell survival-associated signaling, thereby promoting DNA damage repair (54).

Furthermore, TAM-associated hypoxia and paracrine signals may induce cell cycle arrest, providing time for DNA damage repair. HIF-1α has been shown to regulate p21 (CIP1) expression and induce G1 arrest (55). From a radiobiological perspective, G1 arrest gives tumor cells additional time for DNA damage repair, indirectly facilitating the process. However, direct evidence for this specific mechanism is limited. This is especially true when compared to the IL-6 and lactate pathways mentioned above. Therefore, this process represents a potential link in TAM-mediated promotion of DNA damage repair that warrants further investigation.

#### 3.1.2 Maintenance of cancer stem cell properties

CSCs possess stem cell-like self-renewal capacity and robust tumor-initiating ability. Extensive evidence indicates that CSCs promote tumor initiation, progression, invasion, metastasis, and recurrence. They are closely associated with radioresistance and treatment failure (11). TAMs can also directly mediate radioresistance by maintaining CSC-like properties through the paracrine secretion of various signaling molecules.

TAM-derived IL-6 is an important cytokine for the maintenance of CSC-like properties. TAM-secreted IL-6 can activate the JAK/STAT3 signaling pathway in tumor cells, which in turn activates the Wnt/β-catenin signaling pathway. The JAK/STAT3 and Wnt/β-catenin pathways act synergistically to maintain CSC-like properties (56). Similarly, TAM-derived IL-10 can activate JAK1/STAT1 and NF-κB signaling. They cooperatively activate Notch1, thereby promoting CSC-like properties in non-small cell lung cancer (NSCLC) cells (56).

TAMs can also secrete various chemokines to participate in the maintenance of CSC-like properties. For example, M2-like TAMs can secrete CCL20, which activates interleukin-1 receptor-associated kinase-1 (IRAK1) and its downstream NF-κB1/2 in anaplastic thyroid cancer stem cells, subsequently promoting CXCL5 secretion. CXCL5 maintains CSC-like properties through autocrine signaling while simultaneously recruiting macrophages and promoting their polarization toward an M2-like functional state through paracrine signaling (57).

In addition, the TGF-β/Wnt signaling axis participates in TAM-mediated maintenance of CSC properties. In colorectal cancer, TAMs can secrete TGF-β, promoting HIF-1α expression in tumor cells. This upregulates Tribbles pseudokinase 3 and activates the Wnt/β-catenin signaling pathway, thereby promoting CSC-like properties in colorectal cancer (58). This suggests that TAM-mediated regulation of CSC-like properties may be linked to hypoxia-associated signaling pathways. Radiotherapy exacerbates local hypoxia in the TME. In this environment, HIF-1α-related signaling may cooperate with TAM-mediated stemness maintenance to synergistically mediate tumor radioresistance.

Interestingly, in multiple tumor types, CSCs also recruit macrophages into the TME by secreting various soluble factors and promote their reprogramming toward immunosuppressive TAMs. This establishes a positive feedback loop between TAMs and CSCs that ultimately enhances tumor cell tolerance to radiotherapy and other therapeutic modalities (11, 56).

### 3.2 Remodeling of a pro-survival tumor microenvironment

In contrast to the aforementioned mechanisms, this section elucidates indirect regulation at the TME level. TAMs drive post-irradiation TME remodeling through aberrant angiogenesis, ECM remodeling, EMT, and metabolic reprogramming. Consequently, they establish a supportive niche for the survival, invasion, metastasis, and recurrence of residual tumor cells (7, 14).

#### 3.2.1 Hypoxia and aberrant vasculature formation

Aberrant angiogenesis and lymphangiogenesis represent critical mechanisms of TAM-mediated TME remodeling. TAMs can secrete factors such as VEGF, placental growth factor (PlGF), and MMP-9 to drive tumor angiogenesis and lymphangiogenesis (24, 59). Specifically, VEGFA and VEGFC are key regulatory factors for angiogenesis and lymphangiogenesis, respectively. They bind to their respective endothelial cell surface receptors, VEGFR-2 and VEGFR-3, stimulating the proliferation and migration of vascular or lymphatic endothelial cells to form new blood vessels and lymphatic vessels (60). In addition to soluble factors, TAM-derived exosomes can transfer miR-155-5p and miR-221-5p to endothelial cells, thereby promoting tumor-associated neovascularization (59).

However, under the dysregulated signaling, the newly formed vasculature exhibits structural disorganization, high permeability, and poor pericyte and endothelial cell coverage (61). Consequently, tumor angiogenesis fails to effectively improve oxygen delivery to the TME. Rather, it may exacerbate local hypoxia due to inefficient perfusion. Since oxygen can “fix” IR-induced DNA damage, sustained hypoxia can blunt the cytotoxic effects of radiotherapy and increase the probability of survival of tumor cells in hypoxic regions (25). Simultaneously, aberrant lymphatic vessels may provide conduits for tumor cell dissemination, potentially increasing the risk of metastasis and recurrence (61).

Notably, both HIF-1α and VEGF exert chemotactic effects on macrophages to drive their migration to hypoxic tumor regions. The recruited TAMs subsequently release VEGF, PlGF, and MMP-9, establishing a “hypoxia–HIF-1α/VEGF–TAMs” positive feedback loop that continuously drives aberrant vasculature formation and perpetuates the hypoxic TME (24, 59, 62). Therefore, TAM-mediated aberrant angiogenesis and lymphangiogenesis do not directly alter the intrinsic radiosensitivity of tumor cells. Instead, they orchestrate a post-irradiation pro-survival niche through two main pathways: “inefficient perfusion–hypoxia–attenuation of radiotherapy cytotoxicity” and “lymphatic dissemination routes–increased risk of metastasis and recurrence.”

#### 3.2.2 Extracellular matrix remodeling and epithelial–mesenchymal transition

ECM remodeling diminishes the physical resistance to tumor cell migration and invasion by altering the structural architecture of the peritumoral tissue, thereby indirectly affecting radiotherapy efficacy. Studies demonstrate that in TAM–tumor cell co-culture models, TAMs can upregulate MMP2 and MMP9 expression through a TNF-α-dependent mechanism, thereby enhancing ECM protein degradation (11). During this degradation process, MMPs also release or activate matrix-bound VEGF to foster aberrant angiogenesis. Together, these processes generate a permissive environment for tumor cell infiltration and dissemination into surrounding tissues (61, 63). Furthermore, EMT serves as a critical catalyst for tumor invasion and metastasis. TAM-derived TGF-β can induce EMT in tumor cells to enhance their invasiveness and metastatic potential (62). Unlike the hypoxic niche and aberrant vasculature described earlier, which mediate radioresistance by influencing the oxygen enhancement effect, the contribution of ECM remodeling and EMT to radioresistance is more context-dependent. These mechanisms primarily act by remodeling the peritumoral tissue architecture to facilitate tumor cell invasion and metastasis. Consequently, they impede long-term tumor control following radiotherapy. In other words, ECM remodeling and EMT likely represent delayed radioresistance mechanisms. Their detrimental effects predominantly manifest during the TME remodeling phase over extended time scales post-irradiation (64).

#### 3.2.3 Metabolic reprogramming and metabolic competition

Through glucose metabolism, lactate metabolism, amino acid metabolism, and lipid metabolism, TAM-mediated metabolic reprogramming and metabolic competition profoundly reshape the post-irradiation TME. Among these, glucose and lactate metabolism primarily drive radioresistance by establishing a hypoxic niche and disrupting the redox homeostasis of tumor cells. Conversely, amino acid and lipid metabolism primarily mediate tumor cell immune evasion by maintaining TAM immunosuppressive functional states.

Regarding glucose metabolism, TAMs can promote the formation of a hypoxic, glucose-depleted, and acidic TME through paracrine signaling and metabolic competition. Studies demonstrate that TAM-derived TNF-α can upregulate the expression of glycolysis-related genes, including Slc2a1, Hk2, and lactate dehydrogenase A (LDHA), in tumor cells. This shifts tumor cell metabolism toward aerobic glycolysis (the Warburg effect). Simultaneously, TAMs can activate the AMPK/PGC-1α signaling pathway to enhance their own mitochondrial biogenesis and respiratory function. This allows them to compete with tumor cells for oxygen and further exacerbate tumor hypoxia (65). This metabolic competition acts synergistically with the aforementioned vasculature-mediated hypoxic niche, thereby blunting the oxygen enhancement effect of radiotherapy and diminishing the radiosensitivity of the tumor tissue (25).

In classical Hodgkin lymphoma (cHL), a lactate metabolic coupling exists between TAMs and Reed-Sternberg (HRS) cells. Lactate produced by TAM glycolysis is exported via MCT4, while HRS cells import this lactate through MCT1. This upregulates mitochondrial outer membrane subunit 20 and activates NF-κB. This activation subsequently upregulates PGC-1α and NRF-1 to promote oxidative phosphorylation (OXPHOS) and bolster antioxidant capacity (66). These findings suggest that TAMs can modulate mitochondrial function through glycolytic products such as lactate, thereby enhancing tumor radioresistance. However, this mechanism is primarily derived from the cHL context. Its generalizability to other tumor types requires further investigation.

Lactate accumulation in the TME and granulocyte-macrophage colony-stimulating factor (GM-CSF) can induce high expression of Arg1 and glutaminase in TAMs, depleting key nutrients such as L-arginine (L-Arg) and glutamine (67–69). Simultaneously, ornithine and polyamines produced by Arg1-associated metabolism can promote TAM polarization toward an M2-like functional state (68). These amino acid metabolic alterations can modulate the functional states of TAMs and other immune cells and may provide a metabolic basis for the formation of an immunosuppressive TME (67, 68).

Lipid metabolic reprogramming primarily consolidates the immunosuppressive TME by maintaining TAM pro-tumorigenic functional states. In hypoxic, nutrient-deprived, and acidic microenvironments, TAMs acquire energy through increased fatty acid uptake, fatty acid oxidation (FAO), and cholesterol metabolism (69, 70). Studies indicate that M2-like macrophages typically exhibit upregulated expression of the lipid scavenger receptor CD36. CD36 promotes macrophage uptake and intracellular deposition of tumor cell-derived lipids and provides substrates for FAO and OXPHOS through lysosomal lipolysis, thereby supporting the maintenance of M2-like functional states (71). Simultaneously, long-chain fatty acids, as intermediates of FAO, can bind to peroxisome proliferator-activated receptors (PPARs), further promoting FAO and CD36 expression, forming a positive feedback loop that consolidates the M2-like functional state (72).

### 3.3 Attenuation of radiotherapy-induced antitumor immune responses

In recent years, extensive evidence has demonstrated that the antitumor effects mediated by radiotherapy are not entirely determined by its cytotoxic actions, such as induced DNA damage and tumor cell death. Instead, they are also regulated by the post-irradiation immune microenvironment. These immune effects are not limited to the irradiated site but can also influence adjacent tissues and distant, non-irradiated lesions (73). Therefore, the efficacy of radiotherapy-induced immune activation in eradicating tumor cells depends largely on the dynamic equilibrium between pro-inflammatory antitumor signals and immunosuppressive signals within the post-treatment TME. In this balance, TAMs orchestrate an immunosuppressive TME through multiple mechanisms, including suppression of antigen presentation, attenuation of CTL and NK cell function, and promotion of immunosuppressive cell enrichment. Consequently, TAMs antagonize radiotherapy-induced antitumor immune responses, reducing radiotherapy efficacy, and mediating the development of radioresistance.

#### 3.3.1 Restricted antigen presentation

DCs are among the most critical antigen-presenting cells (APCs). They can capture, process, and present tumor antigens to activate CD8^+^ T cells or CD4^+^ T cells, thereby inducing antitumor immune responses (24). However, immunosuppressive TAMs can interfere with this process. TAMs can highly express Arg1, depleting L-Arg and attenuating the metabolic activity and functional integrity of DCs (67). Simultaneously, TGF-β and IL-10 secreted by TAMs can downregulate the expression of MHC class II molecules and co-stimulatory molecules (CD40, CD80, CD86, etc.) on DC surfaces, inhibiting DC maturation and antigen-presenting capacity (62). Additionally, although macrophages themselves possess the capacity for antigen uptake, processing, and presentation, this capacity is highly dependent on the local TME status. Studies have shown that under the therapeutic pressure of radiotherapy and immunotherapy, TAMs may shift toward immunosuppressive functional states such as Spp1^+^ or Arg1^+^, manifesting as restricted antigen-presenting capacity (74).

#### 3.3.2 Restricted effector immune cell function: CTLs and NK cells

CTLs are the core effector cells of the adaptive antitumor immune response following radiotherapy (73). However, TAMs can not only suppress T cell activation and proliferation but also directly attenuate the cytotoxic function of already-activated CTLs and induce their apoptosis. Immunosuppressive TAMs can secrete TGF-β, which interferes with T cell receptor (TCR) signaling and broadly suppresses T cell activation. By downregulating STAT4 expression, TGF-β inhibits Th1 differentiation, thereby limiting CTL activation and proliferation (75). TAM-derived IL-10 can also downregulate the function and expression of antigen processing-associated transporters (TAPs), subsequently downregulating MHC class I expression and making it difficult for CTLs to recognize residual tumor cells following radiotherapy (76).

At the metabolic level, the lactate-accumulating, hypoxic, glucose-depleted TME co-shaped by TAMs and tumor cells can significantly impair CTL function, creating an immunosuppressive environment (70, 74). In mouse models, Arg1^+^ TAMs can deplete L-Arg, which is critical for T cell proliferation and cytotoxicity (67). Simultaneously, nitric oxide (NO) derived from some TAMs can interfere with TCR–MHC interactions through nitrosylation modifications, thereby impairing antigen recognition and cytotoxic capacity of CTLs. Notably, evidence regarding the ability of macrophage L-Arg and NO metabolism to limit CTL function is primarily derived from mouse models or *in vitro* experiments. Their regulatory effects on CTLs in human tumors require further investigation. In comparison, the high expression of immune checkpoint ligands such as PD-L1 has more clearly established clinical relevance to T cell functional suppression in human tumors (77).

PD-L1^+^ TAMs and tumor cells can suppress CTL function through the PD-L1/PD-1 axis. This process can also be mediated by TAM-derived exosomes, which serve as carriers of PD-L1. These exosomes bind to PD-1 on the surface of CD8^+^ T cells and then inhibit CD8^+^ T cell proliferation and activation (78). Simultaneously, CD80 expressed by TAMs can bind with high affinity to CTLA-4 on T cell surfaces, competitively limiting CD28 signaling and thereby suppressing CTL activation and cytotoxic function (79). Furthermore, FasL^+^ TAMs can induce CTL apoptosis through the FasL/Fas axis, further attenuating antitumor immune responses (5).

In addition to CTLs, NK cells are also important effector cells participating in antitumor immune responses following radiotherapy. Human M2-like TAMs can induce NK cells to upregulate the inhibitory receptor CD85j/ILT2 by secreting TGF-β, reducing their IFN-γ production and impairing their antibody-dependent cellular cytotoxicity (ADCC) function (80).

#### 3.3.3 Enrichment of immunosuppressive cells

Tregs are an important immunosuppressive cell population closely associated with tumor cell immune evasion. TAMs can secrete chemokines such as CCL17 and CCL22, which bind to CCR4 on the surface of Tregs, recruiting Tregs into the TME (7, 10). Simultaneously, TGF-β and IL-10 secreted by TAMs promote Treg differentiation, expansion, and functional stability (24). Furthermore, immune checkpoints play important roles in Treg expansion, differentiation, and maturation, including the PD-1/PD-L1 and CTLA-4/CD80 pathways (81).

Notably, enriched Tregs can secrete immunosuppressive cytokines such as IL-10, TGF-β, and IL-35, reducing APC function and suppressing effector T cell responses. They can also constitutively express CTLA-4, downregulating CD80/CD86 expression on APCs and attenuating CD28-dependent co-stimulatory signaling. Consequently, this process inhibits T cell activation (82). Therefore, TAM-mediated Treg enrichment can restrict the activation of the APC–T cell axis following radiotherapy, making it difficult to sustain radiotherapy-induced antitumor immune responses.

### 3.4 Mechanistic hierarchy and context-dependence of TAM-mediated radioresistance

In summary, TAM-mediated radioresistance is not determined by a single mechanism or a fixed TAM subtype. Instead, it represents the combined effect of enhanced intrinsic tumor cell radioresistance, pro-survival TME remodeling, and suppression of antitumor immunity (7, 11, 14, 73).

These effects can be broadly organized into three interrelated tiers: (1) At the level of tumor cell radiotherapy injury response, TAMs primarily modulate DNA damage repair, cell survival, and stemness maintenance. Therefore, these processes are more directly linked to enhanced tumor cell radiotherapy tolerance (11, 51–53). (2) At the level of pro-survival TME remodeling, TAMs orchestrate a pro-tumorigenic TME through hypoxia maintenance, aberrant vasculature formation, matrix remodeling, and metabolic competition. This indirectly blunts radiotherapy efficacy (7, 14). (3) At the level of antitumor immune regulation, TAMs primarily influence whether radiation-induced immunogenic cell death (ICD) can be converted into effective antitumor immune responses. The role is particularly prominent in immunologically active tumors or in the context of combined radiotherapy and immunotherapy (73, 83, 84).

These three tiers are not independent but can intersect through nodes such as hypoxia, HIF-1α, VEGF, TGF-β, IL-10, and exosomes. For example, hypoxia-induced HIF-1α can participate in cell cycle arrest, aberrant angiogenesis, and metabolic reprogramming (55). TGF-β and IL-10 can participate in CSC-like property formation and immunosuppressive TME formation (56, 58, 75). Additionally, exosomes can deliver molecules such as miRNAs, participating in DNA repair, aberrant angiogenesis, metabolic reprogramming, and CTL suppression (54, 59, 78).

Furthermore, the specific manifestations of TAM-mediated radioresistance are markedly context-dependent. Tumor type can influence the origin and functional states of TAMs (15, 85). For example, NRP1^+^ TAMs are more highly expressed in clear cell renal cell carcinoma and influence radioresistance through VEGF-mediated angiogenesis, cathepsin-mediated ECM remodeling, and CTL functional suppression. In contrast, NRP2^+^ TAMs are more highly expressed in cutaneous melanoma and influence radioresistance through MMP-mediated matrix remodeling, lymphangiogenesis, and immune regulation. Similarly, the spatial location of TAMs within the tumor influences their functional states and the primary mechanisms through which they mediate radioresistance. TAMs residing in hypoxic or necrotic regions are more susceptible to hypoxia, lactate accumulation, and tissue injury/repair signals, and may exhibit functional states associated with pro-angiogenesis, tissue repair, and immunosuppression (7, 14, 15). Studies have shown that following immunotherapy or radiotherapy, Spp1^+^/Arg1^+^ macrophages tend to accumulate in necrotic tumor regions in mouse tumors and are associated with restricted T cell activation. Instead, Cxcl9^+^ macrophages predominantly accumulate at the tumor margin, exhibiting antigen-presenting and pro-inflammatory functional states (74). Therefore, the mechanisms by which TAMs mediate radioresistance are not fixed, nor can they be simply attributed to a specific TAM subtype. Rather, they should be understood in the context of tumor type, spatial location, radiotherapy regimen, and temporal changes following treatment.

## 4 Multi-tiered mechanisms by which TAMs mediate radiosensitization

Corresponding to the pro-radioresistant effects described above, TAM-mediated radiosensitization can also be understood from three interrelated levels: tumor cell-intrinsic response regulation, TME remodeling, and immune regulation, mainly corresponding to the mechanisms shown in the green section on the right side of **Figure 2**. Under specific radiation doses, fractionation schedules, and immunological contexts, certain TAMs can exhibit pro-inflammatory responses, enhanced antigen presentation, immune activation, and vascular regulatory functions, thereby augmenting radiation-induced tumor cell damage and antitumor immune responses (15, 18).

### 4.1 Enhancement of intrinsic tumor cell radiosensitivity

Radiotherapy primarily kills tumor cells through direct effects and indirect effects mediated by ROS. Under the oxidative stress and inflammatory signaling induced by radiotherapy, certain TAMs may upregulate iNOS, promoting the production of NO and RNS. These molecules act synergistically with radiation-generated ROS to amplify oxidative and nitrosative stress in tumor cells. This synergy ultimately leads to DNA damage and mitochondrial apoptosis, promoting radiosensitization within a specific time window (86, 87). However, studies have shown that in gastric cancer, squamous cell carcinoma, and hepatocellular carcinoma, tumor malignancy is positively correlated with iNOS activity. Therefore, the iNOS/NO axis cannot be simply viewed as an antitumor or radiosensitizing pathway. Its effects on radiotherapy responses may also be influenced by tumor type, the cellular source of iNOS/NO, NO concentration in the TME, and the duration of exposure (87).

In addition, TAMs formed under exogenous or therapeutic stimulation can enhance radiosensitivity by inhibiting DNA damage repair pathways. In an *in vitro* model of glioblastoma, M1-like TAMs induced by IFN-γ and LPS can secrete pro-inflammatory cytokines such as TNF and IL-1β. These cytokines induce the phosphorylation of STAT1 and subsequently suppress STAT3. This leads to downregulation of DNA repair enzymes such as O6-methylguanine-DNA methyltransferase (MGMT) and N-methylpurine-DNA glycosylase (MPG), thereby impairing the capacity of GBM cells to repair radiation-induced damage (88). Similarly, TAM-derived exosomes can also participate in radiosensitization. For example, in HPV^+^ HNSCC, M1-like TAMs induced by IFN-γ and LPS may secrete exosomes enriched in miR-20b. These exosomes inhibit CCND1 expression in tumor cells, thereby suppressing DNA damage repair and enhancing radiosensitivity (89). Importantly, these studies merely demonstrate that exogenously stimulated inflammatory TAMs possess mechanistic radiosensitizing potential. They do not directly prove that radiotherapy alone can induce the same TAM functional states and exosomal changes *in vivo*. Therefore, these mechanisms should be more appropriately understood as the theoretical basis for targeting TAM functional reprogramming to enhance radiotherapy efficacy and overcome radioresistance.

In summary, the iNOS/NO axis, along with soluble factor- and exosomal miRNA-mediated inhibition of DNA damage repair and cell cycle regulation, suggests that TAMs in specific functional states can enhance intrinsic tumor cell radiosensitivity under certain conditions. However, these sensitizing effects are not universal properties of all TAMs but depend on the TAM functional state, local NO and ROS levels, and the post-irradiation time window.

### 4.2 Improvement of vascular, oxygenation, and metabolic microenvironment

Beyond directly influencing tumor cell DNA damage repair, TAMs may also indirectly alter tumor radiosensitivity by regulating vascular structure, tissue oxygenation, and metabolic status.

The oxygenation status of tumor tissue is an important determinant of radiotherapy efficacy. Oxygen can stabilize radiation-induced DNA damage through the “oxygen fixation effect,” whereas hypoxia reduces the indirect cytotoxic effects mediated by ROS (25). The influence of TAMs on tumor vasculature and oxygenation status is markedly context-dependent. Angio-TAMs primarily mediate radioresistance by promoting aberrant angiogenesis and hypoxia formation (15, 90). In contrast, under conditions of suppressed pro-angiogenic signaling and specific immune activation, tumor vasculature may undergo normalization-like remodeling accompanied by improved perfusion and oxygenation (91). Currently, the evidence base for the former is stronger, whereas the latter requires further validation.

Studies in a mouse melanoma model demonstrated that, in a pro-inflammatory microenvironment induced by immunotherapy, M1-like macrophages can participate in tumor vascular normalization. The increase in M1-like macrophages was accompanied by reduced tumor vessel density, enlarged vessel lumens, increased pericyte coverage, improved perfusion, and reduced hypoxic areas (91). Although this study was not a radiotherapy model, these findings suggest that under specific immune activation conditions, TAMs may optimize TME oxygenation by improving vascular structure and perfusion. These changes may provide an indirect mechanistic basis for radiosensitization through enhanced oxygen fixation and ROS-associated damage (25).

Beyond changes in vascular structure and oxygenation, TAM-associated metabolic reprogramming can also alter the post-irradiation TME. Pro-inflammatory TAMs typically exhibit enhanced glycolysis, accompanied by activation of iNOS, NOX2, and mitochondrial ROS-related pathways. These changes alter the levels of metabolic signals such as NO, ROS, RNS, and lactate. In this process, NOX2-derived ROS can participate in oxidative reactions within phagosomes and intracellular redox signaling, contributing to the maintenance of the inflammatory metabolic state of TAMs (86, 92, 93). However, this effect is markedly time-dependent. Studies have shown that sustained secretion of ROS and RNS may drive the TME into a state of high oxidative stress, promoting TAM polarization toward an M2-like functional state (94).

### 4.3 Amplification of radiotherapy-induced antitumor immune responses

The immune effects of TAMs following radiotherapy are not static but exhibit marked time dependence. Radiotherapy can induce ICD in tumor cells, promoting tumor antigen exposure and releasing DAMPs and cytosolic DNA signals (33–35). These signals are particularly important in the early antitumor immune response following irradiation and can activate the cGAS-STING signaling pathway, driving certain TAMs toward functional states associated with inflammatory responses and antigen presentation (36, 37).

Within this time window, certain pro-inflammatory TAMs themselves function as effector immune cells and can directly kill tumor cells through cytotoxic mechanisms, phagocytosis, and ADCC. Furthermore, pro-inflammatory TAMs can recruit and activate NK cells and CTLs through antigen presentation and the secretion of chemokines such as CXCL9 and CXCL10, as well as pro-inflammatory cytokines such as IFN-γ, TNF-α, and IL-12. This process enhances short-term antitumor immune responses following radiotherapy and indirectly contributes to tumor cell killing (11, 56). Interestingly, IFN-γ is an important signaling factor that mediates interactions among NK cells, T cells, APCs, and macrophages (83, 95). In a pro-inflammatory environment containing IFN-γ, CXCL10^+^ macrophages are more readily maintained. This may enhance the inflammatory activation function of TAMs, thereby contributing to further amplification of antitumor immune responses during the early post-irradiation phase (11).

Therefore, within the early inflammatory window induced by radiotherapy, TAMs can serve as critical cellular nodes linking tumor damage signals, antigen presentation, and effector lymphocyte recruitment, participating in the amplification of radiotherapy-mediated antitumor immune responses. However, whether this process can be converted into effective tumor suppression depends on whether subsequent immunosuppressive TME remodeling occurs, as well as the timing and extent of such remodeling (11, 24).

### 4.4 Mechanistic hierarchy and context dependence of TAM-mediated radiosensitization

Overall, TAM-mediated radiosensitization is not simply equivalent to M1-like polarization. Instead, it represents an integrated regulatory outcome driven by TAMs in distinct functional states under appropriate radiation doses, post-irradiation time windows, and immunological contexts. This outcome involves coordinated effects on intrinsic tumor cell radioresistance, vascular oxygenation, and antitumor immune responses (15, 59, 87, 91, 96).

Among these mechanisms, NO- and RNS-mediated enhancement of oxidative and nitrosative stress, as well as inhibition of DNA damage repair, are more directly related to intrinsic tumor cell radiosensitivity. However, the relevant evidence is largely derived from *in vitro* co-culture models under exogenous stimulation and cannot directly demonstrate that radiotherapy *in vivo* produces the same effects. These findings are more appropriately regarded as a theoretical basis for targeting TAM functional reprogramming to enhance radiotherapy efficacy (88, 89). In contrast, improvements in vascular oxygenation, metabolic reprogramming, and amplification of antitumor immunity are more dependent on the local tumor TME and post-irradiation time window (56, 96). Therefore, TAMs should not be simply defined as radioresistant or radiosensitizing cell populations, but should be understood as a functionally plastic cell population existing along a dynamic continuum. Future studies should integrate scRNA-seq, spatial multi-omics, and functional validation to define the TAM functional states involved in radiosensitization and the therapeutically exploitable time windows (14, 15).

## 5 Therapeutic strategies targeting TAMs to overcome radioresistance: evidence base, limitations, and rationale for combination therapy

The core rationale for targeting TAMs in combination with radiotherapy is not simply to deplete TAMs or induce a uniform pro-inflammatory phenotype. Instead, these strategies aim to intervene in key resistance-associated functions following irradiation. These functions include myeloid cell recruitment, TAM survival, immunosuppression, impaired phagocytosis, and restricted antigen presentation (18, 40, 41, 62).

It is important to emphasize that different strategies vary substantially in their evidence base for combination with radiotherapy and in their translational maturity. Some agents have entered clinical studies or received regulatory approval for specific indications. However, this does not mean that these agents have demonstrated benefit when combined with radiotherapy (97–99). Therefore, this section distinguishes among direct evidence for combination with radiotherapy, evidence from concurrent chemoradiotherapy settings, radiotherapy-related preclinical mechanistic evidence, and indirect evidence derived primarily from non-radiotherapy contexts. The translational status, radiotherapy relevance, and major limitations of representative strategies are summarized in **Table 2**.

**Table 2:** Translational status of representative TAM-targeted and myeloid-directed strategies relevant to radiotherapy resistance.

Evidence category	Strategy	Agent or platform	Target	Clinical status	Radiotherapy relevance and key limitation
Direct trial incorporating radiotherapy	Phagocytosis checkpoint modulation	Evorpacept (ALX148)	CD47–SIRPα axis	Phase II; HPV-associated oropharyngeal cancer; active, not recruiting; NCT05787639	Neoadjuvant SBRT plus evorpacept and pembrolizumab. The single-arm, small-sample design limits attribution of benefit; a radiosensitizing effect remains to be established.
Direct trial incorporating chemoradiotherapy	APC and myeloid activation	APX005M (sotigalimab)	CD40	Phase II; esophageal and GEJ cancers; completed; NCT03165994	Neoadjuvant APX005M plus concurrent chemoradiation. The pilot design and limited TAM-specific endpoints restrict mechanistic attribution.
Clinical development without radiotherapy combination	TAM survival and recruitment blockade	LY3022855 (IMC-CS4)	CSF1R	Early Phase I; borderline resectable pancreatic adenocarcinoma; completed; NCT03153410	Mechanistically relevant to TAM survival and myeloid remodeling. Not designed as a trial combining radiotherapy or chemoradiotherapy; radiosensitizing benefit remains unproven.
Clinical development without radiotherapy combination	TAM survival blockade	PLX3397 (pexidartinib)	CSF1R	Phase II; recurrent glioblastoma; terminated; NCT01349036	Evaluated in recurrent glioblastoma after prior radiotherapy and temozolomide, but not as a radiotherapy-combination trial. Termination was due to a business decision; radiosensitizing benefit remains unproven.
Clinical development without radiotherapy combination	TAM survival blockade	Vimseltinib	CSF1R	Phase III; tenosynovial giant cell tumor; active, not recruiting; NCT05059262	Shows that CSF1R can be clinically targeted. Does not involve radiotherapy or chemoradiotherapy; evidence from tenosynovial giant cell tumor should not be extrapolated to radiotherapy-based oncology combinations.
Clinical development without radiotherapy combination	TAM and monocyte recruitment blockade	BMS-813160	CCR2 and CCR5	Phase Ib/II; colorectal and pancreatic cancers; completed; NCT03184870	Mechanistically relevant to radiotherapy-associated myeloid-cell recruitment and suppression. Clinically tested without radiotherapy or chemoradiotherapy; radiotherapy-combination benefit remains unproven.
Clinical development without radiotherapy combination	TAM and monocyte recruitment blockade	CCX872-B	CCR2	Phase Ib; pancreatic adenocarcinoma; completed; NCT02345408	Mechanistically relevant to radiotherapy-associated monocyte and TAM recruitment. Evaluated with FOLFIRINOX rather than radiotherapy or chemoradiotherapy; radiosensitizing benefit remains unproven.
Clinical development without radiotherapy combination	Myeloid and TAM functional reprogramming	IPI-549 (eganelisib)	PI3Kγ	Phase I/Ib; advanced solid tumors; unknown status; NCT02637531	Mechanistically relevant to radiotherapy-associated myeloid suppression and TAM/MDSC reprogramming. Clinically evaluated as monotherapy or in combination with nivolumab; radiotherapy-combination benefit remains unproven.
Engineered myeloid-cell platform without radiotherapy design	Engineered myeloid-cell platform	CT-0508 and CT-0525	HER2-targeted CAR-macrophage and CAR-monocyte	Phase I; HER2-positive solid tumors; unknown status; NCT04660929 and NCT06254807	No radiotherapy- or chemoradiotherapy-containing trial design was identified. CMC, trafficking, persistence, and phenotypic stability remain key translational barriers.

This table summarizes representative TAM-targeted and myeloid-directed therapeutic strategies with potential relevance to radiotherapy resistance. Trials were classified according to whether radiotherapy or chemoradiotherapy was directly incorporated into the treatment design, or whether radiotherapy relevance was mainly mechanistic or indirect. “Radiotherapy-combination benefit remains unproven” or “radiosensitizing benefit remains unproven” indicates that the listed study either did not include radiotherapy or chemoradiotherapy as a treatment component or did not evaluate radiosensitization as a dedicated clinical endpoint. Clinical trial status was recorded according to ClinicalTrials.gov at the time of manuscript revision.

### 5.1 Conceptual framework and intervention timing for TAM-targeted therapy during radiotherapy

Following irradiation, tumor cell damage can promote the release of DAMPs and the accumulation of cytosolic DNA. These signals can activate type I interferon responses and inflammatory cascades through pathways such as cGAS-STING, thereby remodeling the TME (34, 36). In this context of immune activation, if TAMs retain phagocytic and antigen-presenting capacities, enhancing these functions may help promote T-cell-mediated antitumor responses (40, 41). However, when myeloid cell infiltration, hypoxia, lactate accumulation, and reparative efferocytosis are prominent, TAMs may instead become more involved in immunosuppression and tissue repair (18, 23, 26, 40). Therefore, post-irradiation TAM dynamics should not be interpreted as a simple early pro-inflammatory phase followed by a late reparative phase. Instead, they should be evaluated in the context of radiation dose, fractionation schedule, tumor type, and immune background.

Accordingly, TAM-targeted therapy should not follow a fixed temporal sequence. Instead, the choice of intervention should be guided by the predominant functional state of TAMs following radiotherapy. When TAMs are oriented toward phagocytosis, antigen processing, and antigen presentation, strategies that enhance their antitumor functions may be appropriate. Conversely, when TAMs are oriented toward pro-tumorigenic myeloid replenishment, immunosuppression, or reparative responses, recruitment and survival blockade or functional reprogramming may be more suitable (18, 40, 41). However, this framework remains influenced by radiation dose, fractionation schedule, sampling time, and tumor type. It therefore requires further validation in models that more closely reflect clinical radiotherapy regimens (18).

### 5.2 Targeting TAM recruitment, survival, and functional reprogramming

Recruitment and survival blockade, as well as functional reprogramming, represent two complementary strategies for TAM-directed interventions combined with radiotherapy. The former primarily limits pro-tumorigenic myeloid replenishment. The latter focuses on restoring or enhancing TAM phagocytosis, antigen processing, antigen presentation, and cooperation with T cells (18, 40, 41). Given the marked heterogeneity and dynamic nature of TAMs, these strategies should not be simply interpreted as TAM depletion or the induction of a uniform M1-like phenotype (22). Therefore, recruitment or survival blockade may not always be beneficial. These approaches may inadvertently impair myeloid cells involved in post-irradiation antigen presentation or normal tissue repair.

The CSF-1/CSF-1R axis is a key pathway regulating TAM recruitment and the function of tumor-infiltrating myeloid cells. Studies in glioblastoma indicate that chemotactic signals, including CSF-1/CSF-1R, CXCR4/CXCL12, and HGF/MET, participate in TAM recruitment. Targeting these pathways may therefore reduce TAM infiltration and attenuate immunosuppression (100). In solid tumor models, the CSF1R inhibitor GW2580 reduced the recruitment of tumor-infiltrating myeloid cells, including TAMs and monocytic MDSCs. It also decreased the expression of genes associated with pro-angiogenic and immunosuppressive functions (101). Furthermore, PLX3397 slowed tumor growth and remodeled the TME in a recurrent GBM mouse model (102). Notably, in a GBM xenograft model, the CSF1R inhibitor PLX3397 blocked the differentiation of irradiation-recruited myeloid cells into pro-tumorigenic macrophages. When combined with radiotherapy, this intervention extended mouse survival (18). However, this evidence remains confined to specific preclinical models.

The CCL2/CCR2 axis represents another important target for recruitment blockade. CCL2 promotes the recruitment of TAMs and MDSCs via CCR2. This process remodels the immunosuppressive TME and contributes to tumor invasion and distant metastasis (103). Beyond its effects on myeloid cell survival and functional polarization, this axis regulates the tissue homing and immunosuppressive activity of regulatory T cells. In addition, it promotes the survival, growth, invasion, and metastasis of CCR2-positive tumor cells (104). Nevertheless, multiple cell types continuously produce CCL2. Its expression is jointly regulated by inflammatory cytokines, hypoxia, and various signaling pathways. As a result, the therapeutic efficacy of CCL2/CCR2 blockade alone remains inconsistent (103). Clinically, evidence supporting CCL2/CCR2-targeted therapy remains limited. For instance, carlumab demonstrated poor single-agent efficacy. Similarly, the positive signals observed with PF-04136309 were derived primarily from a chemotherapy-combination study in pancreatic cancer. However, direct evidence for combining these agents with radiotherapy remains lacking (98, 99).

Functional reprogramming focuses on restoring the antitumor effector capacity of TAMs. In non-radiotherapy settings, the combination of IFN-γ with TLR agonists can induce antitumor macrophage activation. In addition, combining TLR7/8 agonists with fatty acid oxidation (FAO) inhibitors can enhance TAM phagocytosis and antitumor function through metabolic reprogramming (105, 106). In the context of radiotherapy-based combinations, PI3Kγ inhibition reprogrammed the TAM response to irradiation-induced apoptotic tumor cells. This intervention shifted TAMs toward antigen presentation and enhanced CD8+ T-cell-dependent tumor control (40). Another study developed a nanomodulator, designated Ft-E64/Hf@Lipo, which incorporated E64 and a hafnium-based radiosensitizing component. This nanomodulator enhanced radiation-induced antigen generation and inhibited excessive lysosomal antigen degradation in TAMs. As a result, it increased MHC-I expression and CD8+ T-cell activation (41). Therefore, these strategies should be selected according to radiotherapy timing, the predominant functional state of TAMs, and the maturity of available evidence (18, 40, 41).

### 5.3 Combining radiotherapy with immune checkpoint and phagocytosis checkpoint blockade

The rationale for combining radiotherapy with immune checkpoint blockade (ICB) lies in counteracting radiation-induced immune escape. Radiotherapy can induce tumor cell death and promote antitumor immunity. However, this process can also trigger escape responses, including PD-L1 upregulation, immunosuppressive cell recruitment, and immunosuppressive cytokine release (84). Therefore, PD-1/PD-L1 blockade is not merely an additional T-cell-directed therapy in radiotherapy-based combinations. Instead, it may help attenuate post-irradiation immunosuppressive feedback. In brain metastases from NSCLC, the optimal timing, efficacy, and safety of combining radiotherapy with immunotherapy remain under investigation (107). Similarly, in hepatocellular carcinoma, radiotherapy combined with immune checkpoint inhibitors has shown some clinical benefit. However, both therapeutic efficacy and treatment-related adverse events may be influenced by the specific combination modality (83).

Notably, PD-1/PD-L1 blockade may exert effects not only through T-cell restoration but also through TAMs. Studies indicate that both murine and human TAMs can express PD-1. Elevated PD-1 expression is associated with reduced phagocytic capacity against tumor cells. Blocking the PD-1/PD-L1 axis can enhance macrophage phagocytosis and suppress tumor growth in a macrophage-dependent manner (108). However, the effects of ICB on TAMs may be model-dependent. In a pancreatic ductal adenocarcinoma model, anti-PD-1 therapy did not further enhance PI3Kγ inhibition-associated efferocytosis (40). In contrast, in other preclinical models, radiotherapy combined with PD-1/TIGIT blockade enhanced abscopal antitumor effects and immune memory. This effect was accompanied by increased interactions between pro-inflammatory macrophages and CD8+ T cells (109). These findings suggest that the efficacy of radiotherapy combined with ICB may depend on the degree of synergy between T-cell restoration and TAM functional remodeling.

By contrast, phagocytosis checkpoint blockade more directly targets macrophage phagocytic function. CD47 is the prototypical “don’t-eat-me” molecule. The CD47/SIRPα axis represents a therapeutically relevant phagocytosis checkpoint (110). In a colorectal cancer model, SIRPα blockade enhanced the antitumor efficacy of high-dose hypofractionated radiotherapy. This effect was accompanied by increased infiltration of activated CD8+ T cells and reductions in MDSCs and TAMs. Further combination with anti-PD-1 therapy enhanced long-term adaptive immune memory (111). Similarly, in a small cell lung cancer (SCLC) model, radiotherapy combined with CD47 blockade enhanced local antitumor activity. It also induced abscopal effects that were macrophage-dependent but T-cell-independent (112). These findings suggest that phagocytosis checkpoint blockade can convert local radiation-induced damage into macrophage-mediated local and systemic antitumor responses.

The clinical translation of these combination strategies, however, requires caution. The therapeutic benefit of combining radiotherapy with ICB may vary depending on tumor type and immune context. For instance, pMMR/MSS colorectal cancer typically shows a limited response to PD-1/PD-L1 monotherapy. This observation indicates that combination strategies require careful patient selection based on immune context (113). In addition, the normal tissue safety and systemic toxicity of CD47/SIRPα blockade combined with radiotherapy remain to be evaluated (110, 112). Therefore, future studies should not focus exclusively on tumor regression or T-cell infiltration. They should also incorporate comprehensive indicators, including TAM phagocytosis, antigen presentation, myeloid immunosuppression, abscopal effects, and normal tissue safety.

### 5.4 TAM depletion and macrophage-related auxiliary strategies

Direct depletion of TAMs represents a relatively straightforward intervention. Clodronate liposomes can deplete TAMs in preclinical models. This depletion is typically accompanied by reductions in tumor vascular density and tumor growth (114). In a squamous cell carcinoma model, this strategy reduced both TAMs and splenic macrophages. This reduction was accompanied by decreased tumor volume, increased apoptosis, and lower vascular density. Metastasis, however, was not significantly reduced (115). These findings indicate that macrophage depletion can attenuate certain pro-tumorigenic support functions. However, it should not be regarded as a strategy capable of comprehensively blocking tumor progression.

Selectivity represents an additional limitation. For instance, an engineered clodronate liposome platform improved the targeting and *in vivo* imaging of M2-like macrophages. However, it also depleted M2-like macrophages in normal liver tissue (116). Consequently, TAM depletion is more appropriate for mechanistic validation or as an auxiliary intervention in specific contexts. It should not be regarded as a broadly applicable radiosensitization strategy.

Macrophage-based platforms can also serve as vehicles for drug delivery. Engineered macrophages, macrophage-derived exosomes, and macrophage membrane-coated nanoparticles have all been explored for tumor-targeted delivery (117). Local irradiation can enrich TAMs in perivascular regions. This enrichment facilitates nanodrug extravasation and intratumoral accumulation. Conversely, TAM depletion attenuates this delivery advantage (118). Therefore, in certain combination strategies, TAMs are not merely therapeutic targets. They may also function as auxiliary nodes to facilitate drug delivery. However, whether these platforms can be reliably translated into TAM-associated radiosensitization benefits requires further validation.

Beyond pharmacological and cell-based platforms, radiotherapy techniques may also influence the conditions for combining TAM-targeted therapy, ICB, or cellular therapies. The Bragg peak and lower exit dose of proton therapy improve dose distribution and reduce irradiation of normal tissues outside the target volume (119). In a CT26 colorectal cancer model, proton irradiation induced intratumoral interferon-related signaling and increased immune cell infiltration. This finding provides experimental support for its combination with ICB (45). Similarly, in a medulloblastoma model, FLASH proton irradiation reprogrammed tumor macrophage lipid metabolism. This reprogramming reduced PPARγ and Arg1 expression. It also promoted a pro-inflammatory macrophage state and enhanced CAR-T cell infiltration and activation (47). Consequently, TAM-related combination strategies should not be designed exclusively around pharmacological or cellular platforms. Instead, they should also incorporate radiotherapy parameters, including irradiation technique, dose rate, and dose distribution. However, the mechanistic basis, translational pathways, and clinical evidence for FLASH-RT require further clarification (49).

### 5.5 Translational evidence and safety boundaries for combining radiotherapy with TAM-targeted strategies

Evaluating the translational value of TAM-targeted strategies requires more than demonstrating their intrinsic antitumor activity. For radiotherapy-based combinations, the key issue is whether these strategies can improve TAM function after irradiation and thereby enhance local tumor control, augment abscopal immune effects, or improve survival. Therefore, even strategies that have entered clinical development should be validated in studies specifically designed to assess radiotherapy-based combinations.

Based on the available literature, relevant strategies can be broadly categorized into three groups. The first category includes preclinical strategies with direct evidence for combination with radiotherapy. For example, studies on PI3Kγ inhibition and Ft-E64/Hf@Lipo suggest that enhancing TAM-mediated processing of irradiation-induced apoptotic tumor cells, antigen presentation, and cooperation with T cells may improve radiotherapy-associated antitumor effects (40, 41). However, this evidence is still mainly derived from animal models.

The second category consists of myeloid-targeting agents that have entered clinical development, but whose supporting evidence is based predominantly on non-radiotherapy settings. For example, the CSF1R inhibitor pexidartinib demonstrated antitumor activity in tenosynovial giant cell tumors (TGCT). However, this evidence does not come from radiotherapy combination studies. In addition, pexidartinib is associated with a risk of mixed or cholestatic hepatotoxicity (97). Similarly, carlumab failed to achieve sustained suppression of free CCL2. The positive signals observed with PF-04136309 also came from combination with FOLFIRINOX chemotherapy, rather than radiotherapy (98, 99).

The third category encompasses strategies with preclinical evidence for radiotherapy-based combinations or platform-based potential, but with insufficient clinical evidence in radiotherapy settings. For instance, CD47/SIRPα blockade has preclinical support for combination with radiotherapy. However, its radiosensitizing effects and TAM-related mechanisms still lack mature clinical validation (111, 112). In addition, CAR-M and macrophage-based delivery platforms are largely supported by evidence from non-radiotherapy settings or early translational studies (117, 120).

A small number of strategies have entered trial designs that incorporate radiotherapy or concurrent chemoradiotherapy. Examples include agents targeting the CD47/SIRPα axis and studies involving CD40 agonists. However, their radiosensitizing effects have not yet been clearly demonstrated. Most myeloid-targeting agents and engineered myeloid cell platforms in clinical development still lack validation in combination with radiotherapy. Representative clinical trials and their translational limitations are summarized in **Table 2**.

Safety considerations also constrain clinical translation. Experience with TAM depletion strategies indicates that direct macrophage elimination may simultaneously affect macrophages in normal tissues (115, 116). In addition, the hepatotoxicity associated with CSF1R inhibitors suggests that systemic myeloid targeting should carefully account for organ toxicity and normal macrophage homeostasis (97). Therefore, future TAM-targeted therapies combined with radiotherapy should not focus exclusively on tumor regression. Instead, they should concurrently evaluate normal tissue injury, myeloid cell homeostasis, and long-term safety.

Future trials should not rely solely on phenotypic markers, such as CD68, CD163, CD206, Arg1, or CD86. Instead, they should incorporate assessments of TAM heterogeneity and dynamic functional states (22, 121). More importantly, trials should evaluate actual TAM functions. These assessments should include monocyte recruitment, apoptotic cell clearance, antigen presentation, the spatial relationship between TAMs and CD8+ T cells, and the overall immunosuppressive status (18, 40, 41). Clinical trial designs should also incorporate key treatment parameters, such as radiation dose, fractionation schedule, treatment sequence, and normal tissue toxicity. Only by linking these indicators to local tumor control, abscopal immune effects, and safety outcomes can researchers determine whether TAM targeting genuinely enhances radiotherapy efficacy, rather than merely altering the post-irradiation immune microenvironment.

## 6 Conclusions and perspectives

Radiotherapy can substantially remodel the TME, and TAMs represent a critical myeloid cell population involved in this process. Current evidence indicates that the M1/M2 model is insufficient to capture the actual functional states of TAMs following irradiation. The same phenotypic markers may correspond to distinct functional profiles depending on tumor type, spatial location, dose and fractionation schedule, and post-irradiation sampling time. Therefore, the influence of TAMs on radiotherapy efficacy should be interpreted in the context of specific tumor types, treatment regimens, and post-treatment time windows. It should not be simply reduced to a fixed dose-polarization framework.

Available studies suggest that TAMs promote radioresistance primarily through three mechanisms. First, TAMs can directly enhance tumor cell tolerance to radiation-induced damage. For example, they facilitate DNA damage repair and maintain cancer stem cell (CSC)-like properties. Second, TAMs can shape a TME that favors the survival and recurrence of residual tumor cells. This process involves hypoxia, aberrant angiogenesis, extracellular matrix remodeling, and metabolic competition. Third, TAMs can attenuate radiotherapy-induced antitumor immune responses. They achieve this by restricting antigen presentation, suppressing cytotoxic T lymphocyte (CTL) and natural killer (NK) cell functions, and promoting the accumulation of immunosuppressive cells. By contrast, TAM-mediated radiosensitization is highly context-dependent. It typically occurs under specific radiation doses, post-irradiation time windows, and favorable immune backgrounds. Furthermore, this effect may rely on intact phagocytic and antigen-presenting capacities or synergy with immunotherapy. Therefore, TAMs do not inherently represent either radioresistance or radiosensitization. Instead, their role depends on their dominant functional state within a specific treatment phase and microenvironmental context.

These insights carry important implications for therapeutic design. Strategies targeting TAMs should not be simply equated with TAM depletion. Furthermore, the induction of an M1-like phenotype should not serve as the sole endpoint. Treatment design should first identify the predominant limiting factors following irradiation. These factors may include sustained myeloid cell recruitment, impaired phagocytosis and antigen presentation, enhanced immunosuppressive signaling, and an increased demand for normal tissue repair. Different limiting factors require distinct interventional strategies. Currently, targets such as CSF-1R, CCL2/CCR2, PI3Kγ, PD-1/PD-L1, and CD47/SIRPα, alongside macrophage-based delivery platforms, show therapeutic potential. However, the maturity of evidence varies considerably across these approaches. Some strategies possess preclinical support for combination with radiotherapy. Others rely primarily on evidence from non-radiotherapy settings. Still others remain at early translational stages. Therefore, antitumor activity observed in non-radiotherapy contexts should not be directly extrapolated to clinical radiosensitization.

Future studies should address three key questions. First, it remains necessary to determine which post-irradiation TAM states genuinely possess therapeutic value. Relying solely on markers such as CD68, CD163, CD206, Arg1, or CD86 is insufficient. Instead, single-cell sequencing, spatial analysis, and functional assays should be integrated. These integrated approaches can clarify whether these cells predominantly mediate tissue repair, immunosuppression, antigen presentation, or pro-inflammatory responses following irradiation. Second, the optimal timing for TAM-targeted interventions remains to be defined. TAMs may play distinct roles during the early and late phases after radiotherapy. Consequently, the same agent may produce divergent outcomes when administered at different time points. Furthermore, the dynamic changes in TAMs during clinically conventional fractionated radiotherapy remain insufficiently characterized. Third, the balance between efficacy and safety should be incorporated into study designs. Macrophages contribute to normal tissue repair and immune homeostasis. Therefore, systemic TAM targeting may introduce additional risks. The hepatotoxicity associated with CSF1R inhibitors and the impact of non-selective macrophage depletion on normal tissue repair both highlight these hazards. They indicate that such combination approaches require careful design.

TAMs lie at the intersection of radiation-induced damage, tissue repair, and antitumor immunity. Future TAM-targeted radiosensitization should not focus solely on altering phenotypic markers or immune infiltration profiles. Instead, these strategies should demonstrate improvements in local tumor control, enhanced abscopal antitumor effects, and prolonged survival. Furthermore, they should achieve these outcomes without substantially increasing normal tissue toxicity. Only when the targets, timing, efficacy endpoints, and safety boundaries are clearly defined can TAM-targeted strategies become a reliable component of radiotherapy-immunotherapy combinations.

## Glossary

Arg1: Arginase 1
CAFs: Cancer-associated fibroblasts
CCL: C-C chemokine ligand
CCR: C-C chemokine receptor
CD47-SIRPα: CD47-signal regulatory protein alpha
cGAS-STING: Cyclic guanosine monophosphate-adenylate synthase-stimulator of interferon genes
CRT: Calreticulin
CSCs: Cancer stem cells
CSF: Colony-stimulating factor
CTLA-4: Cytotoxic T-lymphocyte-associated protein-4
CTLs: Cytotoxic T cells
CXCL: C-X-C motif chemokine ligand
DAMPs: Damage-associated molecular patterns
DCs: Dendritic cells
DDR: DNA damage response
DNA-PKCs: DNA-dependent protein kinase catalytic subunits
ECM: Extracellular matrix
ECs: Endothelial cells
EGF: Epidermal growth factor
EMT: Epithelial-to-mesenchymal transition
EVs: Extracellular vesicles
FAO: Fatty acid oxidation
FLASH-RT: FLASH radiotherapy
HIF: Hypoxia-inducible factors
ICD: Immunogenic cell death
ICIs: Immune checkpoint inhibitors
IFN: Interferon
IL: Interleukin
iNOS: Inducible nitric oxide synthase
JAK: Janus kinase
L-Arg: L-arginine
MDSCs: Myeloid-derived suppressor cells
MHC: Major histocompatibility complex
NF-κB: nuclear factor-κB
NK cells: Natural killer cells
NO: Nitric oxide
NOX2: NADPH oxidase 2
NRF-1: nuclear respiratory factor-1
OXPHOS: oxidative phosphorylation
PD-1: Programmed cell death protein-1
PD-L1: Programmed cell death ligand-1
PlGF: Placental growth factor
RNS: Reactive nitrogen species
ROS: Reactive oxygen species
STAT: Signal transducer and activator of transcription
TAMs: Tumor-associated macrophages
TGF: Transforming growth factor
TME: Tumor microenvironment
TNF: Tumor necrosis factor
Tregs: Regulatory T cells
VEGF: Vascular Endothelial Growth Factor.
